# Prognostic value of surfactant protein D and biochemical markers in BALF and plasma of ARDS patients undergoing mechanical ventilation

**DOI:** 10.5937/jomb0-59884

**Published:** 2026-01-06

**Authors:** Hu Han, Litao Zhang, Zhangshun Shen, Ranliang Hua, Lingling Li, Hui Guo

**Affiliations:** 1 Hebei General Hospital, Department of Emergency, Shijiazhuang, China

**Keywords:** Surfactant protein D, exosomes, bronchoalveolar lavage fluid, ARDS, plasma biomarkers, biochemical predictors, surfaktantski protein D, egzozomi, tečnost bronhoalveolarnog lavaža, ARDS, plazma biomarkeri, biohemijski prediktori

## Abstract

**Background:**

Surfactant protein D (SP-D) and circulating exosomes have emerged as potential biochemical indicators of lung injury severity in acute respiratory distress syndrome (ARDS). This study aimed to evaluate the prognostic value of SP-D levels and selected biochemical parameters in bronchoalveolar lavage fluid (BALF) and plasma among ARDS patients receiving mechanical ventilation.

**Methods:**

A total of 103 mechanically ventilated ARDS patients were enrolled between February 2020 and February 2023. Patients were classified into survival (n = 59) and death (n=44) groups based on 28-day mortality. On the day of diagnosis, SP-D and exosome levels in BALF and plasma, along with pH, lactate, and oxygenation-related indices, were measured and analyzed for prognostic relevance.

**Results:**

SP-D levels in both BALF and plasma were significantly higher in non-survivors (P &lt; 0.001), while exosome levels did not differ significantly. The death group also showed elevated lactate and lower pH levels (P&lt; 0.05). ROC analysis demonstrated high predictive value for SP-D in BALF (AuC=0.804) and plasma (AU C= 0.864), as well as for lactate and oxygenation indices. A combined biomarker model yielded an AUC of 0.883 for predicting 28-day mortality.

**Conclusions:**

SP-D concentrations in BALF and plasma, along with lactate and acid-base markers, serve as valuable biochemical predictors of short-term prognosis in ARDS patients undergoing mechanical ventilation.

## Abstract (sr)

**Uvod:**

Surfaktantski protein D (SP-D) i cirkulišući egzozomi pojavili su se kao potencijalni biohemijski indikatori težine oštećenja pluća kod akutnog respiratornog distresa (ARDS). Cilj ove studije bio je da se proceni prognostička vrednost nivoa SP-D i odabranih biohemijskih parametara u tečnosti bronhoalveolarnog lavaža (BALF) i plazmi kod pacijenata sa ARDS-om koji primaju mehaničku ventilaciju.

**Metode:**

Ukupno 103 mehanički ventilirana pacijenta sa ARDS-om su uključena u istraživanje između februara 2020. i februara 2023. godine. Pacijenti su klasifikovani u grupe preživljavanja (n = 59) i smrti (n=44) na osnovu mortaliteta od 28 dana. Na dan dijagnoze, nivoi SP-D i egzozoma u BALF i plazmi, zajedno sa pH, laktatom i indeksima povezanim sa oksigenacijom, mereni su i analizirani na prognostički značaj.

**Rezultati:**

Nivoi SP-D i u BALF i u plazmi bili su značajno viši kod nepreživelih (P&lt; 0,001), dok se nivoi egzozoma nisu značajno razlikovali. Grupa sa smrtnim ishodom je takođe pokazala povišen nivo laktata i niži nivo pH (P&lt; 0,05). ROC analiza je pokazala visoku prediktivnu vrednost za SP-D u BALF (aUc =0,804) i plazmi (AUC = 0,864), kao i za indekse laktata i oksigenacije. Kombinovani model biomarkera dao je AUC od 0,883 za predviđanje mortaliteta u roku od 28 dana.

**Zaključak:**

Koncentracije SP-D u BALF i plazmi, zajedno sa markerima laktata i acidobazne kiseline, služe kao vredni biohemijski prediktori kratkoročne prognoze kod pacijenata sa ARDS-om koji se podvrgavaju mehaničkoj ventilaciji.

## Introduction

Acute Respiratory Distress Syndrome (ARDS) is a severe, acute, and progressive pulmonary condition marked by extensive alveolar damage and impaired gas exchange [1] [2] [3]. Epidemiological data indicate that ARDS affects up to 10% of the population and carries a mortality rate ranging from 27% to 37%, posing a significant clinical and public health burden [4] [5]. While mechanical ventilation remains the primary supportive therapy to maintain oxygenation and carbon dioxide elimination, it may also induce ventilator-associated lung injury due to alveolar overdistension or oxygen toxicity, thus complicating prognosis [6] [7]. As a result, there is a pressing need for reliable biomarkers to assess disease severity and guide clinical decision-making in ARDS.

Beyond ventilatory parameters, biochemical indicators are increasingly recognized for their potential prognostic value in ARDS. The oxygenation index (PaO_2_/FiO_2_), a physiological marker widely used in clinical practice, is closely correlated with mortality in critically ill ARDS patients [8]. In parallel, circulating exosomes - nanovesicles carrying proteins, nucleic acids, and lipids - are involved in intercellular communication and may reflect the extent of systemic inflammation and alveolar-capillary barrier disruption during ARDS. Surfactant protein D (SP-D), a pulmonary collectin predominantly secreted by alveolar epithelial cells, plays a critical role in innate immune defense and lung homeostasis. Elevated serum levels of SP-D have been associated with increased disease severity and worse outcomes in ARDS [9].

Given these insights, evaluating SP-D and exosome levels in both plasma and bronchoalveolar lavage fluid (BALF) may offer clinically relevant biochemical markers to predict prognosis in ARDS patients. This study was therefore conducted to explore the relationship between the oxygenation index, SP-D and exosome concentrations in BALF and plasma, and 28-day mortality among mechanically ventilated ARDS patients. The results are reported below.

## Materials and methods

### General information

This prospective observational study was conducted from February 2020 to February 2023 and included 103 patients diagnosed with acute respiratory distress syndrome (ARDS) who received mechanical ventilation at our hospital. Patients were divided into two groups based on their 28-day survival: the survival group (n = 59) and the death group (n = 44). There were no statistically significant differences in baseline characteristics between the two groups (P > 0.05), as shown in Table 1. The study protocol was approved by the hospital's ethics committee, and written informed consent was obtained from all patients or their legal representatives.

**Table 1:** General data comparison.

Group	Age (year)	Sex		BMI (kg/m^2^)	Weight (kg)
		male	female		
Survival group<br>(n=59)	52.15±5.66	42 (71.19)	17 (28.81)	22.16±3.16	62.15±6.45
Death group<br>(m=44)	51.26±4.98	29 (65.91)	15 (34.09)	22.23±2.98	61.98±7.15
* χ^2^/t *	0.830	0.328		-0.114	0.126
* P *	0.408	0.567		0.910	0.900
Group	Basic aetiology				
	Severe pneumonia	Multiple inj'ury	Septic shock	Cerebral apoplexy	Severe pancreatitis
Survival group<br>(n=59)	10(16.95)	15(25.42)	12(20.34)	5(8.47)	17(28.81)
Death group<br>(m=44)	12(27.27)	8(18.18)	6(13.64)	4(9.09)	14(31.82)
* χ^2^/t *	2.584				
* P *	0.630				

### Inclusion criteria:

(1) Diagnosis of ARDS based on the consensus definition of the European and American conferences [10];

(2) Age between 19 and 65 years;

(3) No recent history (within three months) of high-dose antibiotic or immunosuppressant use;

(4) Availability of complete clinical and laboratory data

### Exclusion criteria:

(1) Patients who did not complete the entire treatment course at our institution, were transferred to another facility, or discharged prematurely;

(2) Patients with a prior history of respiratory disease treatment or those with recurrent ARDS episodes;

(3) Presence of severe autoimmune diseases;

(4) Diagnosed malignancies;

(5) Death within five days of hospital admission.

### Sample collection:

Peripheral venous blood and bronchoalveolar lavage fluid (BALF) were collected from all participants on the day of diagnosis. Venous blood samples were centrifuged at 2,500 rpm (radius 14 cm) for 10 minutes at room temperature, and the resulting serum was harvested for biochemical analysis. BALF was obtained using fiberoptic bronchoscopy under local anesthesia. The bronchoscope was advanced into the right middle lobe bronchus, where 50 mL of sterile 0.9% sodium chloride solution at 37°C was instilled and then aspirated twice. The retrieved lavage fluid was immediately filtered through sterile gauze to remove mucus and debris, followed by centrifugation at 2,500 rpm (radius 14 cm) for 10 minutes. The supernatant was collected and stored for further analysis.

### Exosome quantification:

Exosome size distribution and concentration in BALF and plasma were measured using nanoparticle tracking analysis (NTA), which detects particles based on Brownian motion and light scattering. The total concentration of exosomes was expressed as particles/mL, and natural logarithmic transformation was applied to the values for statistical analysis.

### SP-D detection:

Surfactant protein D (SP-D) concentrations in both BALF and plasma were quantified using commercially available enzyme-linked immunosorbent assay (ELISA) kits (Roche, Shanghai, China), following the manufacturer's instructions. All samples were measured in duplicate to ensure analytical precision.

### Arterial blood gas and derived indices:

Immediately after diagnosis, arterial blood samples were collected and analyzed using an automated blood gas analyzer (Cobas B123, Roche) to measure pH, arterial oxygen partial pressure (PaO_2_), and the fraction of inspired oxygen (FiO_2_). From these parameters, the following ventilatory indices were calculated:

Plateau pressure-oxygenation index: Pplat X 100 X FiO_2_/PaO_2_

Driving pressure-oxygenation index: P X 100 X FiO_2_/PaO_2_

### Observation indicators

The primary biochemical indicators assessed on the day of diagnosis included SP-D and exosome levels in both BALF and plasma. In addition, arterial blood gas variables (pH, PaO2, lactate) and mechanical ventilation parameters were recorded. The relationship between these biochemical and physiological parameters and the 28-day prognosis was subsequently analyzed.

### Statistical analysis

Data were processed using Statistic Package for Social Science (SPSS) 23.0 software (IBM, Armonk, NY, USA). Categorical variables such as sex and underlying etiology were analyzed by chi-square test. Continuous variables such as age, BMI, exosome content, and SP-D levels were analyzed by t-test. A p-value < 0.05 was considered statistically significant.

## Results

### Comparison of exosome and SP-D levels in BALF

As shown in Table 2, there was no statistically significant difference in the concentration of exosomes in bronchoalveolar lavage fluid (BALF) between the survival and death groups (P > 0.05). In contrast, BALF SP-D levels were markedly higher in the death group compared to the survival group (P < 0.05), suggesting a potential association between elevated SP-D and adverse clinical outcomes.

**Table 2:** Comparison of exosome and SP-D content of BALF.

Group	Exosome (mL^-1^)	SP-D (μmg/mL)
Survival group (n=59)	26.78±1.65	29.26±2.12
Death group (m=44)	26.22±1.23	94.26±5.46
* t *	1.892	-83.500
* P *	0.061	<0.001

### Comparison of plasma exosome and SP-D levels

Similarly, no significant differences in plasma exosome levels were observed between the two groups (P > 0.05). However, plasma SP-D concentrations were significantly elevated in the death group relative to the survival group (P < 0.05), as demonstrated in Table 3.

**Table 3:** Comparison of exosomes and SP-D contents in plasma.

Group	Exosome (mL^-1^)	SP-D (μg/mL)
Survival group (n=59)	25.54±2.12	19.26±1.46
Death group (m=44)	25.03±2.31	42.12±6.45
* t *	1.162	-26.373
* P *	0.248	<0.001

### Comparison of arterial blood gas parameters

As shown in Table 4, arterial oxygen partial pressure (PaO_2_) and carbon dioxide partial pressure (PaCO_2_) did not differ significantly between groups (P > 0.05). However, patients in the death group exhibited significantly lower arterial pH values and higher lactate concentrations compared to those in the survival group (P < 0.05), indicating more profound metabolic derangements and tissue hypoxia.

**Table 4:** Comparison of arterial blood gas indexes.

Group	pH value	PaO_2_ (mmHg)	PaCO_2_ (mmHg)	Lac (mmol/L)
Survival group (n=59)	7.44±0.12	83.85±34.12	44.45±15.26	2.11±1.02
Death group (m=44)	7.38±0.13	78.44±46.26	45.11±15.68	2.87±1.26
* t *	2.422	0.683	-0.215	-3.381
* P *	0.017	0.496	0.831	0.001

### Comparison of mechanical ventilation parameters

Mechanical ventilation parameters are presented in Table 5. Compared with the survival group, the death group showed significantly lower oxygenation index (PaO_2_/FiO_2_) values, alongside increased oxygen index, plateau pressure-oxygenation index, and driving pressure-oxygenation index (all P < 0.05). These findings suggest more severe respiratory impairment and ventilatory dependence in non-survivors.

**Table 5:** Comparison of mechanical ventilation parameters.

Group	Oxygenation index	Oxygen index	Platform oxygen pressure index	Drive oxygen pressure index
Survival group (n=59)	181.55±56.12	7.35±2.12	12.66±6.12	8.77±3.56
Death group (m=44)	124.62±66.45	17.88±5.16	28.45±9.26	18.44±6.97
* t *	4.706	-14.171	-10.407	-9.181
* P *	<0.001	<0.001	<0.001	<0.001

### Predictive value of SP-D, blood gas indicators, and ventilation parameters

**Figure 1:**
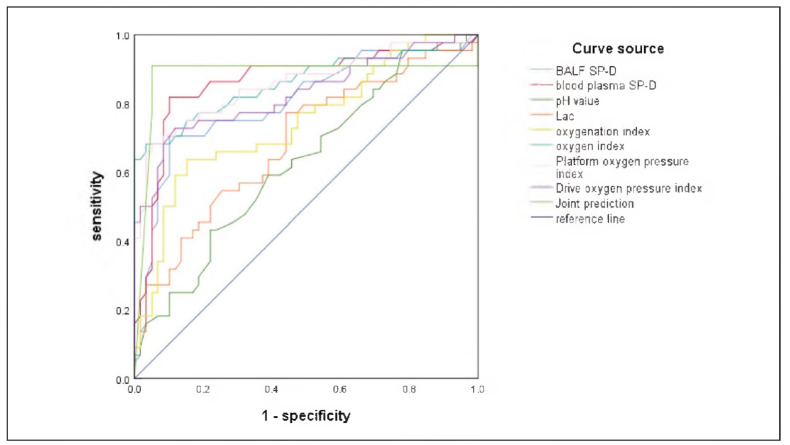
Path diagram of mediation analysis. (Note: Figure 1 represents the mediation analysis path diagram of inflammatory factors on the relationship between glycemic abnormalities and disease severity. A-C represent the mediating effects of IL-6, WBC and hs-CRP, respectively).

Receiver Operating Characteristic (ROC) curve analysis was performed to evaluate the prognostic performance of each parameter. As shown in Table VI and Figure 1, the area under the curve (AUC) for BALF SP-D and plasma SP-D were 0.804 and 0.864, respectively, both indicating strong predictive value. Additional parameters with notable predictive ability included oxygen index (AUC = 0.861), plateau pressure-oxygenation index (AUC = 0.857), and driving pressure-oxygenation index (AUC = 0.828).

The combination of these high-performing indicators (AUC > 0.750) yielded an overall AUC of 0.883, surpassing the predictive value of any single parameter. This combined biomarker approach demonstrated high sensitivity (0.909) and specificity (0.849) for predicting 28-day mortality in ARDS patients.

## Discussion

Acute Respiratory Distress Syndrome (ARDS) is a life-threatening pulmonary condition typically induced by severe infections, trauma, or exposure to harmful inhalants, leading to diffuse alveolar injury and impaired gas exchange [11] [12]. Although its pathogenesis is multifactorial and not yet fully elucidated, ARDS is largely driven by an exaggerated systemic inflammatory response. This cascade results in the recruitment of inflammatory cells to the lungs, excessive cytokine release, increased alveolar-capillary permeability, and subsequent pulmonary edema [13] [14] [15]. These changes compromise lung compliance and oxygenation capacity, frequently resulting in hypoxemia and multi-organ dysfunction. Mechanical ventilation remains a critical supportive intervention; however, its efficacy varies and may itself contribute to ventilator-induced lung injury. Hence, the identification of reliable biochemical and physiological markers for prognosis is crucial to guide therapeutic strategies and improve patient outcomes.

Among candidate biomarkers, surfactant protein D (SP-D) has gained considerable attention. SP-D is a collagen-containing C-type lectin produced primarily by alveolar epithelial type II cells and plays an integral role in pulmonary host defense and surfactant homeostasis [16]. In the present study, we found significantly higher SP-D levels in both bronchoalveolar lavage fluid (BALF) and plasma in patients who succumbed to ARDS, compared to survivors. This suggests that elevated SP-D reflects heightened alveolar epithelial injury and systemic inflammation, both of which are linked to poorer prognosis. During mechanical ventilation, lung tissue may be subjected to cyclic overdistension and oxidative stress, resulting in increased release of SP-D into the alveolar space and systemic circulation [17]. High SP-D levels may therefore indicate a maladaptive inflammatory response and impaired alveolar-capillary integrity. Previous studies have also proposed that SP-D contributes to epithelial cell apoptosis by modulating cas- pase-related pathways, further exacerbating lung injury and worsening clinical outcomes [18].

Conversely, our data showed no statistically significant difference in exosome concentrations between the survival and death groups. While exosomes are recognized carriers of various bioactive molecules—including proteins, RNAs, and lipids— that mirror the functional state of their parent cells, their prognostic value in ARDS remains uncertain. It is possible that the molecular composition of exosomes, rather than their concentration alone, may be more predictive of patient outcomes. Additionally, differences in exosome origin, isolation techniques, and quantification methods may contribute to inconsistencies in their assessment. Therefore, future research should focus on characterizing the molecular cargo of exosomes and elucidating their functional roles in the pathophysiology of ARDS.

Biochemical analysis of blood gas parameters revealed that patients in the death group had significantly lower pH and elevated lactate levels. These findings are consistent with metabolic acidosis secondary to systemic hypoperfusion and cellular hypoxia, reflecting inadequate tissue oxygenation in the context of severe lung injury [19]. Lactate accumulation and acidemia have long been associated with poor prognosis in critically ill patients, including those with ARDS.

Additionally, we evaluated a panel of ventilatory indices including the oxygenation index (PaO_2_/FiO_2_), oxygen index, plateau pressure-oxygenation index, and driving pressure-oxygenation index. Lower oxygenation index and higher values of the other indices in non-survivors indicate more severe impairment in pulmonary function and increased dependence on mechanical ventilatory support. These metrics serve not only as clinical severity indicators but also as indirect reflections of alveolar-capillary damage and altered lung mechanics [20].

ROC analysis demonstrated that SP-D levels in both BALF and plasma, as well as lactate, pH, and oxygenation-related indices, each possess independent prognostic value. Notably, combining multiple high-performing biomarkers—specifically those with AUC values greater than 0.75—resulted in the highest predictive accuracy for 28-day mortality (AUC = 0.883, sensitivity = 0.909, specificity = 0.849). These findings highlight the importance of a multiparameter biochemical approach to ARDS risk stratification. Early assessment of SP-D, lactate, and ventilatory indices may facilitate the identification of patients at increased risk of mortality, thereby enabling more timely and targeted interventions.

In summary, this study demonstrates that elevated SP-D levels in BALF and plasma, together with key biochemical and ventilatory parameters, are significantly associated with poor prognosis in mechanically ventilated ARDS patients. These biomarkers may serve as valuable tools for early risk assessment and personalized management in critical care settings.

## Dodatak

### Funding

This work was supported by the A Comparative Study on the Effects of High-Concentration Oxygen Inhalation and Good PEEP Levels on Lung Injury (20170034).

### Conflict of interest statement

All the authors declare that they have no conflict of interest in this work.

## References

[1] Gragossian A, Siuba M T (2022). Acute Respiratory Distress Syndrome. Emerg Med Clin N Am.

[2] Bos L, Ware L B (2022). Acute respiratory distress syndrome: causes, pathophysiology, and phenotypes. Lancet.

[3] Jing P, Wu C, Du C, Zhou L, Gu L (2024). Predictive value of plasma sICAM-1 and sP-Selectins in the risk of death in patients with acute respiratory distress syndrome. J Med Biochem.

[4] Yehya N, Smith L, Thomas N J, Steffen K M, Zimmerman J, Lee J H, et al (2023). Definition, Incidence, and Epidemiology of Pediatric Acute Respiratory Distress Syndrome: From the Second Pediatric Acute Lung Injury Consensus Conference. Pediatr Crit Care Me.

[5] De Luca D, Tingay D G, van Kaam A H, Courtney S E, Kneyber M, Tissieres P, et al (2022). Epidemiology of Neonatal Acute Respiratory Distress Syndrome: Prospective, Multicenter, International Cohort Study. Pediatr Crit Care Me.

[6] Spinelli E, Mauri T, Beitler J R, Pesenti A, Brodie D (2020). Respiratory drive in the acute respiratory distress syndrome: pathophysiology, monitoring, and therapeutic interventions. Intens Care Med.

[7] Abrams D, Schmidt M, Pham T, Beitler J R, Fan E, Goligher E C, et al (2020). Mechanical Ventilation for Acute Respiratory Distress Syndrome during Extracorporeal Life Support. Research and Practice. Am J Respir Crit Care.

[8] Hueda-Zavaleta M, Copaja-Corzo C, Miranda-Chávez B, Flores-Palacios R, Huanacuni-Ramos J, Mendoza-Laredo J, et al (2022). Determination of PaO2/FiO2 after 24 h of invasive mechanical ventilation and DeltaPaO2/FiO2 at 24 h as predictors of survival in patients diagnosed with ARDS due to COVID-19. Peerj.

[9] Agustama A, Surgean V A, Utariani A (2022). Correlation of Surfactant Protein-D (SP-D) Serum Levels with ARDS Severity and Mortality in Covid-19 Patients in Indonesia. Acta Med Acad.

[10] Kogan A, Segel M J, Ram E, Raanani E, Peled-Potashnik Y, Levin S, et al (2019). Acute Respiratory Distress Syndrome following Cardiac Surgery: Comparison of the American-European Consensus Conference Definition versus the Berlin Definition. Respiration.

[11] Saguil A, Fargo M V (2020). Acute Respiratory Distress Syndrome: Diagnosis and Management. Am Fam Physician.

[12] Bittner E, Sheridan R (2023). Acute Respiratory Distress Syndrome, Mechanical Ventilation, and Inhalation Injury in Burn Patients. Surg Clin N Am.

[13] Huppert L A, Matthay M A, Ware L B (2019). Pathogenesis of Acute Respiratory Distress Syndrome. Semin Resp Crit Care.

[14] Zhang J, Ge P, Liu J, Luo Y, Guo H, Zhang G, et al (2023). Glucocorticoid Treatment in Acute Respiratory Distress Syndrome: An Overview on Mechanistic Insights and Clinical Benefit. Int J Mol Sci.

[15] Kuebler W M (2017). The Flow-Dependent Transcription Factor KLF2 Protects Lung Vascular Barrier Function in Acute Respiratory Distress Syndrome. Am J Respir Crit Care.

[16] Elmore A, Almuntashiri A, Wang X, Almuntashiri S, Zhang D (2023). Circulating Surfactant Protein D: A Biomarker for Acute Lung Injury?. Biomedicines.

[17] Upreti S, Prusty J S, Kumar A, Samant M (2023). Identification of SARS-CoV-2 Spike Protein Inhibitors from Urtica Dioica to Develop Herbal-Based Therapeutics Against COVID-19. World J Trad Chinese.

[18] Murugaiah V, Agostinis C, Varghese P M, Belmonte B, Vieni S, Alaql F A, et al (2020). Hyaluronic Acid Present in the Tumor Microenvironment Can Negate the Pro-apototic Effect of a Recombinant Fragment of Human Surfactant Protein D on Breast Cancer Cells. Front Immunol.

[19] Zhang H, Li Z, Zheng W, Zhang L, Yang T, Xie K, et al (2022). Risk stratification of patients with acute respiratory distress syndrome complicated with sepsis using lactate trajectories. Bmc Pulm Med.

[20] Wu S H, Kor C T, Chi S H, Li C Y (2023). Categorizing Acute Respiratory Distress Syndrome with Different Severities by Oxygen Saturation Index. Diagnostics.

